# Targeted Delivery of Specialized Pro-Resolving Mediators (SPMs) for Improved Treatments of Inflammatory Diseases and Cancer

**DOI:** 10.3390/pharmaceutics18091048

**Published:** 2026-08-23

**Authors:** Adeola Aminu, Zhenjia Wang

**Affiliations:** 1 Department of Pharmaceutical Sciences, Division of Drug Delivery and Pharmaceutical Bioengineering, School of Pharmacy and Pharmaceutical Sciences, University at Buffalo, SUNY, Buffalo, NY 14215, USA; adeolaam@buffalo.edu; 2 Department of Biomedical Engineering, School of Engineering and Applied Sciences, and Jacobs School of Medicine and Biomedical Sciences, University at Buffalo, SUNY, Buffalo, NY 14260, USA

**Keywords:** inflammation, specialized pro-resolving mediators, delivery, cancer

## Abstract

Acute and chronic inflammation underlies the pathogenesis of numerous diseases, including autoimmune disorders, atherosclerosis, infections, and cancer. Although non-steroidal anti-inflammatory drugs (NSAIDs) and corticosteroids are widely used to control inflammation, their clinical utility is limited by adverse effects such as gastrointestinal toxicity and immunosuppression. Specialized pro-resolving mediators (SPMs), a family of endogenous lipid mediators derived from omega-3 fatty acids, have emerged as promising therapeutics because they actively promote the resolution of inflammation without suppressing host immunity. However, their clinical translation is hindered by poor chemical stability, rapid metabolic degradation, and short circulation half-lives. To overcome these limitations, a variety of delivery platforms—including liposomes, extracellular vesicles, PLGA nanoparticles, and hydrogels—have been developed to improve SPM stability, pharmacokinetics, and therapeutic efficacy. This review summarizes the cellular targets of SPMs, current delivery challenges, and emerging strategies for cell- and tissue-specific SPM delivery. We also discuss future opportunities for targeted SPM therapies in the treatment of inflammatory diseases and cancer.

## 1. Introduction

Acute inflammation occurs in response to tissue injury or infections, and its complete and timely resolution is critical for restoration of tissue homeostasis [1,2]. However, if inflammation is not resolved on time, it leads to a wide range of acute and chronic pathologies including delayed wound healing, atherosclerosis, autoimmune diseases, and cancer [3,4]. For example, inflammation becomes overwhelming, resulting in a cascade of adverse events involving immune cell activation and transmigration that consequently compromises inflammation resolution processes. Currently, therapies including non-steroidal anti-inflammatory drugs and corticosteroids are clinically used to treat inflammation-related diseases, but they are limited in use because of many side effects, such as gastrointestinal toxicity and immunosuppression [5,6,7,8]. This highlights the urgency to develop new therapeutics. As inflammation resolution is a natural self-resolving process that restores tissue homeostasis, there has been increased research interest in inflammation resolution with the development of new and effective therapies to treat many inflammatory diseases.

Recent studies [5,6,9,10,11] have shown that acute inflammation is resolved by recently discovered lipid-derived molecules produced in inflammatory tissues. These molecules are called specialized pro-resolving lipid mediators (SPMs). Further studies show that SPMs are produced from ω-3 polyunsaturated fatty acids (PUFAs). Docosahexaenoic acid (DHA) is one of the polyunsaturated fatty acids PUFAs that is metabolized in a sequence of biochemical reactions involving 15-lipoxygenase (15-LOX) and 5-lipoxygenase (5-LOX) to produce D-series Resolvins. Examples are Resolvin D1 and D2 (called RvD1 and RvD2) which are well studied [12,13,14,15,16]. Lipidomic mass spectroscopy studies have identified the biosynthesis pathways of RvD1 and RvD2 and their molecular structures. Their biological functions have been comprehensively investigated and show that they possess potent multifaceted actions [17,18,19]. For example, RvD1 or RvD2 inhibit leukocyte migration, induces neutrophil apoptosis, diminishes the expression of endothelial adhesion molecules, enhances immune cell phagocytosis and decreases the production of proinflammatory cytokines. It was revealed that resolvins act on GPCRs (G-protein coupled receptors), inducing downstream signaling pathways, and their potency is at picomolar levels [17,20]. Other SPMs include Maresins (MaRs) and Protectins (PDs), which are derived from DHA, Resolvin E series (RvEs) derived from eicosapentaenoic acid (EPA) and Lipoxins derived from arachidonic acid (AA).

**Figure 1:**
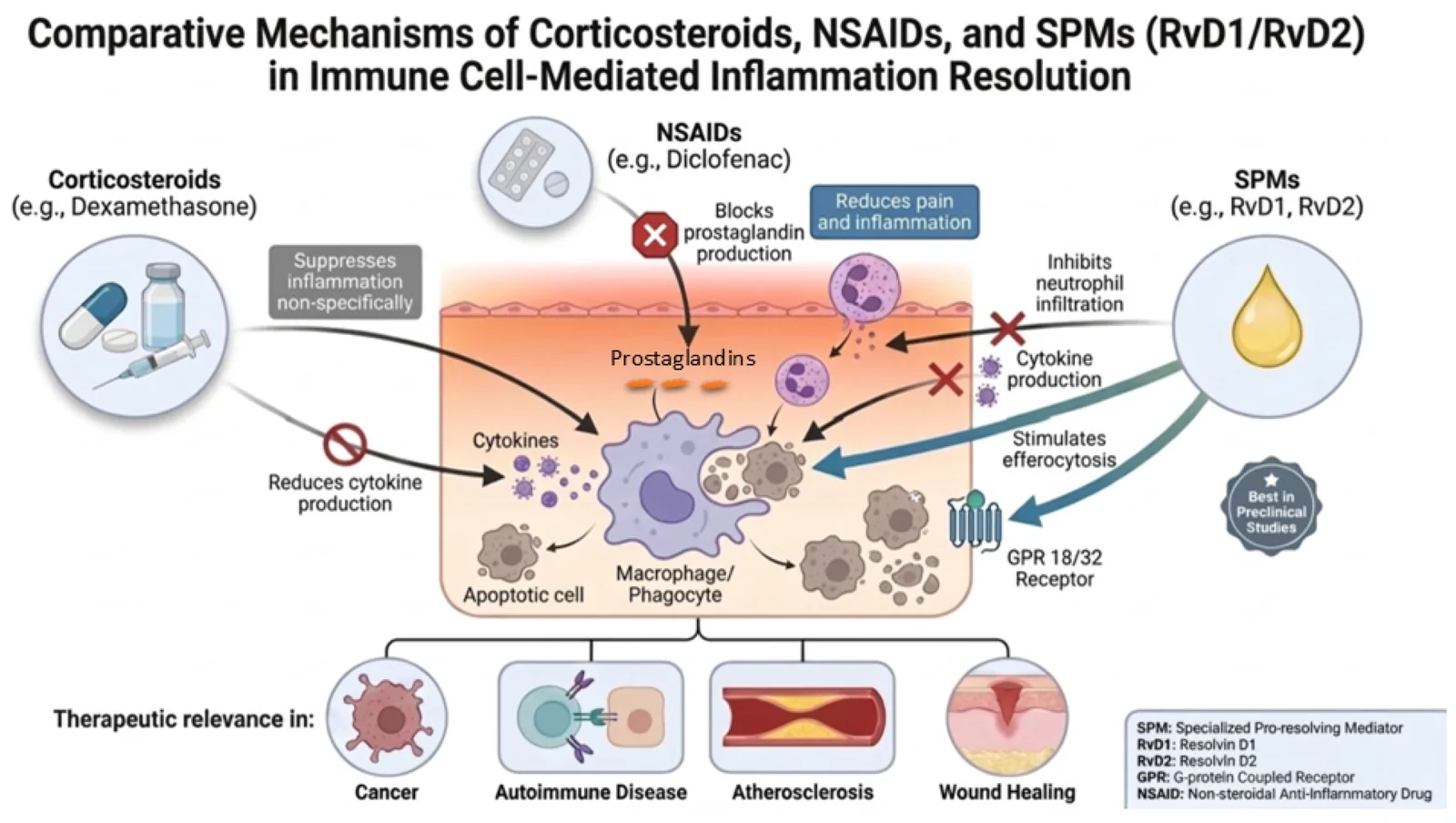
Schematic describes the mechanisms of drugs used in inflammation-related pathologies. NSAIDs inhibit *cyclooxygenase* enzymes that convert arachidonic acid to inflammatory prostaglandins. Corticosteroids bind intracellular glucocorticoid receptors which translocate to the nucleus to inhibit the NF-κB signaling pathway that produces inflammatory cytokines. SPMs, such as RvDs, exert their resolution effects by inhibiting immune cell infiltration, reducing cytokine production and increasing macrophage phagocytosis. Figurelabs.ai was used to generate this figure.

Studies have shown that if inflammation is unresolved, chronic inflammation ensues and leads to a wide range of inflammatory diseases and autoimmune disorders, such as atherosclerosis, stroke, myocardial infarction and rheumatoid arthritis [21,22]. Furthermore, rodent models [23,24,25] and clinical studies [26,27] have demonstrated that targeting inflammation resolution pathways is a novel means to treat inflammatory diseases. Thus, SPMs could be novel therapeutics to treat a wide range of inflammatory diseases and infections [28,29,30]. Figure 1 shows the comparison of mechanisms between NSAIDs, corticosteroids, and SPMs (such as RvD1/2) in treating inflammatory diseases, wound healing and cancer. SPMs are becoming increasingly investigated as alternatives to NSAIDs and corticosteroids [4,6,31]. Their preclinical studies showed prophylactic effects and treatment in infectious disease models, atherosclerotic models, oxidative stress models and several types of cancers [4,32,33]. This has led to increased research interest in their use in treating and managing different pathologies. Figurelabs.ai was used to generate figures in the manuscript. Mechanisms of action of SPMs, NSAIDs, and corticosteroids were provided to the software, and it was repeatedly fed a conceptual comparison in text to generate a draft image which was later edited and finalized in Microsoft PowerPoint software.

At the preclinical level, RvD1 and RvD2 are usually administered intravenously or intraperitoneally in many models. The short circulation time (about 4 h), and their rapid degradation by *eicosanoid oxidoreductase* and *15-prostangladin dehydrogenase* limit the delivery of SPMs to targeted tissues [34,35]. SPMs are unstable in ambient conditions and are prone to degradation by light, oxidation, and temperature. Therefore, it is imperative to design and develop novel delivery systems to increase the tissue targeting of SPMs.

In this review, we will provide an overview of the molecular mechanisms of SPMs for inflammation resolution, and we will discuss how SPMs regulate different types of immune cells in inflammation-related pathologies and cancer. Secondly, we will review the current delivery strategies and approaches to improve the tissue targeting of SPMs. At the end, we will discuss the challenges and opportunities of SPMs as new and novel therapeutics to improve the current treatments for cancer and inflammatory diseases.

## 2. SPMs Regulate Cellular Functions in Inflammatory Diseases

SPMs bind ALX/FPR2 (RvD1, and Lipoxins), ERV1/ChemR23 (RvE1), DRV1/GPR32 (RvD1), DRV2/GPR18 (RvD2), and LGR6 (MaR1) to effect pro-resolving properties with a distinct ligand–receptor specificity, as evidenced by structure activity relationship studies (SAR) [33]. This is followed by the activation of downstream signaling pathways leading to modulation of cellular functions such as reduced production of cytokines and chemokines, reduced neutrophil extracellular traps, increased neutrophil apoptosis, and increased macrophage phagocytosis. Neutrophils, macrophages, monocytes, dendritic cells, T cells and B cells express one or more of the GPCR receptors listed [4,33,36,37]. The target cells and SPMs’ roles in their modulation in inflammation are discussed below.

### 2.1. Neutrophils

Neutrophils are the most abundant immune cells in the human body, constituting about 40–75% of white blood cells, and play a central role in inflammation response [38]. They are rapidly activated in response to inflammatory stimuli and migrate from blood to infectious sites to restrain infections. Neutrophil trafficking is specific and highly regulated by several membrane proteins and cytokine/chemokine gradients [39]. Based on the unique transmigration, neutrophils are explored as drug delivery systems to deliver therapeutics across blood vessel barriers to treat acute respiratory distress syndrome (ARDS) [40] and cancer [41,42].

However, neutrophils themselves may contribute to the development of many pathologies such as cancer, atherosclerosis, neurodegeneration and autoimmune diseases if they are out of control or undergo excessive activation. Their roles in inflammation and resolution pathways have been established [43]. During acute inflammation, they are primarily governed by classical inflammatory chemoattractant GPCR systems (CXCR1/2, BLT1, C5aR1, FPR1, PAFR), which often generate stronger and more amplified signaling than pro-resolving SPM receptors under inflammatory conditions [44]. This could explain why receptor occupancy by SPMs does not always translate to resolution. It has been postulated that the ALX/FPR2 receptor is promiscuous to both SPMs and endogenous pro-inflammatory ligands like serum amyloid A, bacterial formyl peptides, and cathelicidins [44,45].

Post trauma and microbial attack, neutrophils are recruited early to the tissues in a few minutes, actively contributing to the recruitment of other inflammatory mediators, such as cytokines, chemokines and other immune cells [43,46,47]. Their diverse heterogeneity makes them assume a multifaceted role in inflammation and resolution through tissue infiltration, formation of neutrophil extracellular traps (NETs), their apoptosis and efferocytosis by macrophages [47,48]. They are also involved in resolution via the release of soluble molecules like annexin A1, Resolvins and Lipoxins, then finally going into apoptosis followed by macrophage efferocytosis [48]. Their fate and functions are tissue-context specific and can be dependent on cytokine gradients, hypoxia, and interactions with neighboring cells in the stroma. This makes neutrophils a promising therapeutic target to modulate the immune system.

Infectious diseases like COVID-19, chronic inflammation and autoimmune disorders could lead to excessive formation of NETs leading to tissue damage. Using a microfluidic NET-capturing device, the newly discovered Resolvin Ts (RvT1-4) were found to significantly reduce NET formation in PMA-stimulated human whole blood at nanomolar concentrations [32]. A dose- and time-dependent effect was observed with IL-1β-stimulated neutrophils with a 50% NET inhibition at 10 nM. This was later validated in vivo using a murine model of *Staph. aureus* where RvTs reduced neutrophil infiltration, bacterial load, and NET accumulation. Neutrophils phagocytose microbes, e.g., bacteria or yeast, and undergo apoptosis to be later efferocytosed by pro-resolution macrophages. It was observed that RvE1 accelerates neutrophil apoptosis followed by phagocytosis of opsonized *E.coli via* mechanisms, such as activation of BLT1 receptors that boosts reactive oxygen species ROS production, suppression of the ERK-Akt pathway, and activation of caspase-8 and caspase-3 [49].

In post tissue transplantation, there is an early leukocyte-mediated tissue injury, followed by endogenous production of SPMs such as RvD1 that limit the inflammation progression [50]. However, the immune response to tissue grafting could lead to a swarming of neutrophils into the organs so overwhelming that endogenously produced SPMs would not be enough. In a mouse model, RvD1 limited neutrophil infiltration and swarming, thus improving the grafting functions and limiting tissue damage [50]. The results suggest that SPMs could be a therapeutic intervention to prevent transplantation-induced tissue injury.

While lipoxins LXA_4_ and LXB_4_ were found to reduce ROS production in atherosclerosis patients compared to that in healthy individuals, the chronic inflammation and continued failure of inflammation resolution persisted, highlighting insufficient amounts of SPMs in atherosclerosis lesions [51]. This raises questions on dosing frequency for SPMs and the time course to regulate neutrophil infiltration in atherosclerotic lesions. Overall, SPMs modulate neutrophil functions and shift them towards a pro-resolution phenotype, making them strong candidates for a wide range of pathologies associated with neutrophil infiltration, such as kidney injury, chronic obstructive pulmonary disease COPD, and COVID-19.

### 2.2. Macrophages

Macrophages are non-granulocytic myeloid cells that make up 10% in number and about 50% in mass of the total human immune cells [52]. This is due to their abundance across different types of tissues, and higher capacity compared to other immune cells. They are the master phagocytes involved in innate immune responses to infections, and inflammatory responses [4,36]. The target receptors on macrophages for RvD1 and RvD2 are GPR32 and GPR18 respectively, while RvE1 and Maresin 1 (MaR1) target ChemR23/BLT1 and LGR6 receptors respectively [36,53]. Given the central role of inflammatory stimuli in many pathologies, macrophages offer a huge therapeutic potential to be programmed for inflammation resolution [54].

Cigarette smoking impacts macrophage functions that induce chronic obstructive pulmonary disease (COPD) [55]. RvD1 and RvD2 reverse cigarette smoke-induced macrophage dysfunction, offering a non-immunosuppressive strategy to treat chronic inflammatory conditions like COPD and smoke-related lung disease. RvD1 has been found to rescue impaired efferocytosis in vitro and in vivo in a murine atherosclerotic model [56]. Infiltrating macrophages in amyotrophic lateral sclerosis (ALS) become hyperinflammatory due to increased production of IL-1β, IL-6 and TNF-α, contributing to poor prognosis [57]. The inflammatory macrophage-mediated neuronal phagocytosis leads to motor neuron loss in ALS, as approximately 20% of neurons were found engulfed by macrophages. However, RvD1 inhibited this inflammatory process, offering a promising anti-inflammatory therapeutic strategy. Similar anti-inflammatory outcomes were observed in mice treated with RvE1 in an LPS-mediated cardiac injury model with significant reduction in myocardial inflammation [53]. With respect to macrophages, polarization, reduction in cytokine production and increase in phagocytotic or efferocytotic capacity are the typical desired outcomes in numerous pathologies.

### 2.3. Dendritic Cells

Dendritic cells express ChemR23/ERV1, ALX/FPR2, GPR32, and GPR18 receptors like other immune cells. Evidence showed that SPMs modulate dendritic cell maturation and function to reduce inflammation and restore tissue homeostasis [58]. In an experimental autoimmune encephalitis model, RvD1 and the chemically stable analogue 4-(2′-methoxyphenyl)-1-[2′-[N-(2″-pyridinyl)-p-fluorobenzamido]ethyl]piperazine (p-MPPF) resulted in less demyelination and reduced inflammatory infiltration in the CNS—a consequence of inflammation resolution [34]. Their effect on reducing DC activation and antigen presenting capacity shifts them towards a tolerogenic phenotype in encephalitis, a less desirable outcome in the context of cancer treatment.

### 2.4. Monocytes

Monocytes are non-granulocytic myeloid cells involved in innate immune response [52]. They are produced in bone marrow and circulate in blood and migrate into various tissues. Bacteria activate monocytes to induce the host inflammation responses. For example, after lipopolysaccharide (LPS) binds to CD14 and LBP (lipopolysaccharide-binding protein), the TLR4 signaling cascade is activated, leading to increased pro-inflammatory cytokine production [59]. However, the binding of RvD2 to GPR18 receptors decreased the production of IL6, IL8, and TNF-α, and reduced TLR4 expression by 75%. Gu et al. identified that RvD1, RvD2 and MaR1 stimulated increased phosphorylation of glycogen synthase kinase 3β (GSK3β), leading to reduced TLR4-mediated inflammatory response [60]. In multiple sclerosis where the monocytes are hyperinflammatory, they are adhesive and transmigrate to cross the blood brain barrier, leading to poor prognosis. SPMs such as LXA_4_, LXB_4_, RvD1 or PD1 caused a significant reduction in CD69 surface expression, TNF-a, IL-1b, IL-6 and IL-12. The general trend post SPM administration in multiple pathologies was a shift of monocytes from proinflammatory to anti-inflammatory, as evidenced by studies involving ELISA measurement of cytokines and flow cytometry analysis of surface receptors such as surface Ly6C protein, a marker for monocytes [59,61].

### 2.5. T Cells and B Cells

SPMs bind ChemR23/ERV1, GPR32, or ALX/FPR2 receptors expressed on T lymphocytes and B lymphocytes to modulate cellular functions [3,62]. While the innate immune cells are mostly involved in acute inflammation, T lymphocytes -Th1, Th2, Th17 and Treg-play a significant role in chronic inflammation [63]. This is possible through their stimulation by pro-inflammatory cytokines and their constitutive capacity for high cytokine production, amongst which are IL-6, TNF-a, IL17, IL23, IL21, IL-22 and GM-CSF. Using flow cytometry to distinguish cell populations, RvE1 was found to reduce the IL-17+ and ROR gamma+ Th17 cell populations typically observed post polarization and post activation. In addition, the secretion of IL-21, IL2 and CCL20 was reduced post RvE treatment. SPMs such as RvD1 and the intermediate product of DHA to the RvD biochemical pathway 17-HDHA were found to promote spleen derived B cell differentiation into IgM and IgG antibody-producing cells [64]. The study found a significant production of IgM and IgG following treatment with RvD1 and 17-HDHA. Peripheral activated B cells also had a decreased IL6 and IL10 production following exposure to 17-HDHA. RvD1 was found to decrease B cell IgE production via suppression of class switching typical of IgG-, IgA-, and IgE-producing B cells [65]. This was found to reduce the burden of asthma, an inflammation-driven pathology that could be exacerbated following binding of IgE to Fc receptors on mast cells.

### 2.6. Endothelial Cells

The endothelium is a permeable barrier that regulates fluids and nutrients across blood vessels and tissues [66,67,68,69,70]. Inflammation responses cause the activation of endothelial cells and recruitment of leukocytes [11]. Excessive activation of endothelial cells leads to a wide range of diseases, such as atherosclerosis, hypertension, and aneurysms. Therefore, endothelial cells are novel targets to develop therapies. In line with immune cells, endothelial cells also have the capacity to secrete inflammatory cytokines when exposed to inflammatory stimuli. While reports on SPM receptor expressions on endothelial cells are limited, Flak et al. proposed the orphan GPR101 receptor may be responsible for the RvD5_n−3 DPA_ pro-resolving effects on endothelial cells [71]. GPR101 is expressed in human vascular endothelial cells (HUVECs) and has been shown to regulate the endothelial KLF2 transcriptional program, although RvD5 did not significantly alter KLF2 expression in HUVECs in that study [72].

While there is a low level of receptor expression for SPMs, it is possible to deliver SPMs to tissue resident immune cells by targeting inflammatory endothelial cells. Based on the interactions between leukocytes and endothelial cells, neutrophil membrane-derived nanovesicles were developed to deliver anti-inflammatory agents and RvDs to treat lung injury [73,74], stroke [75] and infections [76,77].

## 3. SPMs Regulate Cellular Functions in Cancer

Specialized pro-resolving mediators (SPMs) and their precursors regulate multiple cellular processes relevant to cancer development and progression by acting on both tumor cells and immune cells in the tumor microenvironment [4,78,79]. Rather than functioning solely as anti-inflammatory mediators, SPMs actively regulate leukocyte trafficking, cytokine production, macrophage phagocytosis and efferocytosis, and cellular responses involved in tissue homeostasis [4,79,80]. Collectively, these cell-specific actions suggest that SPMs can regulate cancer progression through coordinated effects on tumor cells, innate and adaptive immune cells, and the inflammatory tumor microenvironment. In this section, SPM effects on selected cell types in the context of cancer are discussed.

### 3.1. Neutrophils

The tumor microenvironment is rich in TGF-β, IL-6, GM-CSF, CXCL1/2/5/8, PGE2, which promote neutrophil trafficking and polarization into N2 neutrophils which promote tumor progression [77,81]. SPMs may reduce tumor-promoting tumor-associated neutrophil (TAN) functions and favor tumor-killing neutrophil phenotypes. In the tumor microenvironment, tumor-associated neutrophils through interactions with cancer cells promote tumor progression instead of killing them. In tandem with their effects in inflammatory diseases, SPMs inhibit the formation of neutrophil extracellular traps (NETs) and promote neutrophil-mediated recruitment of antitumor monocytes [32,80]. Antitumor roles of TANs translate to tumor cytotoxicity through the secretion of protease granules and production of reactive oxygen species (ROS) and myeloperoxidase (MPO) [81]. RvD1 polarized neutrophils into a pro-resolving phenotype in a human papilloma virus model [80]. The polarization led to chemoattraction of monocytes to kill tumor cells in vivo. AT-RvD1 produced by acetylated COX2 and lipoxygenase inhibited neutrophil infiltration in a lung adenocarcinoma model to cause improved lymphocyte antitumor effects [58].

### 3.2. Macrophages

Macrophages attack foreign particles and phagocytose apoptotic and necrotic cells (efferocytosis) [4]. Like many other innate immune cells, macrophages are diverse in phenotypes and functions. The specific phenotype determines the specific function in different pathological states. Examples are M1 macrophages, which are typically called pro-inflammatory, and M2, pro-resolution macrophages. Binding of RvD1 and RvD2 to GPR32 and GPR18 receptors enables macrophages to assume pro-resolution phenotypes, leading to increased efferocytosis, reduced inflammatory cytokine production, and increased anti-inflammatory cytokine production, a desirable outcome in post cancer chemotherapy [4]. Likewise, tumor-associated macrophages (TAMs) shift into a pro-resolving antitumor phenotype post SPMs administration to restore immune surveillance compromised by classical TAMs [58,82]. In an in vitro model designed to investigate the effect of RvDs on TAMs and cancer cell growth, RvD1 and RvD2 blocked the polarization of macrophages into tumor-promoting TAMs, thereby preventing their ability to stimulate cancer cell growth. They do not directly affect cancer cells or already polarized TAMs [54].

Necrotic cells can cause a prostanoid storm that leads to impaired macrophage efferocytosis through p-AMPK signaling, oxidative phosphorylation, and fatty acid oxidation [83]. In a chemotherapy-induced inflammation model, necrotic cells caused aggressive regrowth of surviving tumor cells. However, administration of RvD1 and RvD2 inhibited tumor growth and in some cases shrunk it [4]. This was attributed to improved PS-mediated recognition of cell debris (apoptotic and necrotic cells) by macrophages and subsequent efferocytosis post RvD1 treatment.

### 3.3. Tumor Cells

There are reports of EPA and DHA inducing apoptosis in hematological malignancies cell lines and cancer stem cells at micromolar concentrations via mechanisms involving mitochondrial perturbation, reactive oxygen species ROS formation, lipid peroxidation, lipid raft disruption, inhibition of NF-κB and PI3K/Akt pathways, and immunomodulation [84,85,86]. The outcome of SPM intervention and overall cancer burden is conflicting in the literature when they or their precursors (e.g., EPA and DHA) are used alone. Some reports suggested a 32% reduction in Ki67, the proliferation marker in a phase 2 randomized study of 48 patients with prostate cancer [62,87,88]. Similar results were reported in a clinical trial involving EPA intervention in breast cancer patients [89]. However, this was not corroborated in a colorectal cancer clinical trial, with the authors citing the need for pharmacogenetics analysis to better explain the data [78,87], i.e., the investigation of how the differences in actions of drug metabolizing enzymes—*lipoxygenase*, and *cyclooxygenase* enzymes—impacts the conversion of the precursor to active SPMs.

The Serhan group reported a reduction in the tumor burden with both RvD1 and RvD2 in a chemotherapy-induced inflammation model [4]. While Ki67 marker was not investigated in this study, the authors attributed the actions of RvDs to immunomodulation. In as yet unpublished data from our group, we found that intravenous and intratumoral administration of RvD2 alone without combination with a chemotherapeutic had no effect on tumor growth/volume, highlighting their ineffectiveness as a standalone therapy in Lewis lung carcinoma and melanoma cancer models. In contrast, the Serhan group reported an indirect action of RvD1 and RvD2 in preventing chemotherapy-induced regrowth of tumor cells due to the impact of pro-resolution macrophages [4]. However, the apoptotic tumor cells were generated ex vivo as opposed to in vivo in our study. While similar outcomes would be expected, the nuance in the study design and its impact on SPMs’ efficacy requires further research for translational purposes.

The SPMs’ effect on cancer cells is quite multimodal with respect to mechanisms as it ranges from direct action on receptors like FPRL1 receptor to suppressing angiogenesis and modulation of surrounding immune cells via GPR32, FPR2, and GPR18, as seen with RvD1 and RvD2 [62]. Lee et al. reported that RvD1 through binding to FPR2 and GPR32 inhibits TGF- β1-induced epithelial mesenchymal transition of A549 lung cancer cells to prevent tumor growth and metastasis [90]. Despite conflicting evidence in the literature, we posit that the effect of RvD1 and RvD2 in the tumor microenvironment is immune-cell dependent to increase the phagocytosis of apoptotic and necrotic cancer cells. The potential application of these two SPMs would be in a context where the tumor burden has been reduced via chemotherapy, radiotherapy, or surgery.

### 3.4. Other Cell Types

Evidence of SPMs’ effect on T cells and B cells in cancers is limited in the literature. It is well established that SPMs reduce the constitutive capacity of many immune cells to produce inflammatory cytokines [3,64,65]. Corroborating this with the few studies on inhibitory effects of SPMs (e.g., lipoxin A4 and RvD1) over pro-tumoral cells, such as Breg lymphocytes, and stimulatory effects on antitumoral immune cells, such as Tregs, strengthens the antineoplastic claims [62]. IgM produced naturally by B cells may modulate inflammatory responses to clear precancerous, apoptotic and necrotic cells, contributing to immune regulation and potentially anticancerous effects indirectly [91,92].

Dendritic cells are a special group of immune cells involved in antigen presentation to cytotoxic T cells. Their role spans across immunogenic and tolerogenic responses leading to antitumor immunity or immune tolerance, respectively [93,94]. While the role of dendritic cells in cancer development and progression has been studied, particularly the subset cDC1 and their therapeutic potential, little is known about their modulation by SPMs in the context of improving antitumor immunity [93,94].

SPMs’ effects on the other cell types such as monocytes and endothelial cells in cancer are closely related to their roles and effects in inflammation-related conditions where they play significant roles in chemotaxis and trafficking across tissues. Table 1 and Table 2 below summarize the most targeted cell types in different pathologies and the therapeutic outcomes observed.

## 4. Delivery Systems for SPMs

While SPMs hold significant therapeutic potential, their clinical translation is hampered by issues of stability, delivery, specificity, and disease context. Overcoming these challenges will require both pharmacological innovations and a deeper understanding of resolution biology. This section discusses their delivery challenges and the most common delivery systems used.

SPMs are not only prone to oxidation, heat, and light exposure; they are also rapidly broken down by eicosanoid-metabolizing enzymes contributing to their reduced half-lives [35,53]. RvD1 and RvD2 spontaneously partition into lipid bilayer via hydrophobic interaction and hydrogen bonds [96]. Therefore, it is urgent to develop delivery systems to reach the target tissues and control the release in the inflammatory sites. In the preclinical models, free RvDs are usually administered intravenously repeatedly or intraperitoneally via an osmotic pump, making it challenging in translation [4]. Different delivery systems have been developed to address these challenges for improved pharmacokinetics and reduction in the frequency of dosing [38,76,97,98]. Amongst them are in liposomes, extracellular vesicles/exosomes, hydrogels, nanosheets, etc.

### 4.1. Liposomal Delivery Systems

Liposomes are by far the most common delivery systems that have been employed in SPM delivery. They help improve their stability by preventing rapid oxidation, facilitate sustained release, and could be functionalized for improved tissue targeting [99]. While the precursors of SPMs (such as EPA and DHA) are easily incorporated into liposomes [99], incorporating RvD1 or RvD2 is more challenging given their reduced lipophilicity due to the multiple hydroxyl groups [31,33]. Platelet membrane coating of liposomes were used to improve the stability and increase targeting to injured vasculature and inflamed cardiomyocytes [100]. The targeting effect was further improved upon by incorporating an ROS-responsive linker that releases RvD1 only in high-ROS environments like a reperfused myocardium.

In an osteoarthritis mouse model, liposomes were used as a sustained release system to deliver RvD1 to reduce inflammation and improve joint function [97,98]. With a reported high encapsulation efficiency of 71% and an in vitro sustained release level for up to 11 days in a phosphate-buffered saline solution, there is potential for further optimization. However, many of the loading attempts in the literature using liposomes require the formulations to be used immediately as the packing and sustained release of the liposomes content could not be guaranteed long term—more than 50–75% of the drug is released within the first day [97,98].

### 4.2. Cell Membrane-Derived Nanovesicles

The activation of endothelial cells is a hallmark of inflammatory diseases and the interactions between neutrophils and endothelial cells are features of a wide range of vascular diseases [39]. Gao et al. proposed a novel strategy to make neutrophil membrane nanovesicles to target inflammatory vasculature [73]. In order to target inflammation tissues and bacteria, they developed a novel method to co-load RvD1 and the antibiotic, ceftazidime inside nanovesicles [77]. Their results showed that nanovesicles can specifically target inflamed lung vasculature for the treatment of infection and inflammation caused by *P. aeruginosa*. The advantage of their formulation is that the neutrophil nanovesicles retained integrin β_2_ that can bind to ICAM-1 on inflamed lung endothelium. Therefore, their nanovesicles can target inflammatory sites. In an ischemic brain model, the same group found that neutrophil membrane nanovesicles preferentially accumulated in ischemic brain regions due to neutrophil-derived surface proteins targeting inflamed brain endothelium [75]. Mice treated with nanovesicles loaded with RvD2 showed smaller infarct volumes, improved neurological scores, and reduced blood–brain barrier disruption.

In a similar approach with the addition of a cationic lipid Dimethyldioctadecylammoniumbromide DDAB, neutrophil-derived EVs loaded with DHA, a precursor of Resolvin Ds showed improved targeting of lung tissue in an acute respiratory disease syndrome ARDS model [101]. This concept involves a top-down or hierarchical approach where there is preferential accumulation in inflamed lungs via uptake by inflammatory cells in the lung microenvironment, and improved therapeutic action inside target cells. EVs may carry enzymes involved in lipid mediator biosynthesis, enabling in situ production of anti-inflammatory lipids [102]. Their membrane composition and cargo reflect the parent cell’s inflammatory status, suggesting potential for personalized or engineered EV therapies targeting specific tissues to be used in conditions such as myocardial infarction. The endogenous circulating extracellular vesicles have been highlighted to serve as a delivery system for endogenously produced SPMs amongst other bioactive endogenous metabolites. Extracellular vesicles can carry proteins such as CD5L, an immunomodulatory protein involved in macrophage function, lipid handling, and inflammation resolution [103]. It was proposed that in acute-on-chronic liver failure, the EV cargo becomes defective, weakening the resolution of inflammation as EVs act as carriers of pro-resolving signals, including CD5L and SPM-related lipids.

While the use of neutrophil derived vesicles is emerging, macrophage derived vesicles have been used to making MLP-NVs (Macrophage-Lipidated Peptide Nanovesicles) that were produced by fusing M2-macrophage membranes and a lipidated therapeutic peptide (DOPE-pp-HBSP), loaded with cholesterol-reducing agent simvastatin [104]. The delivery platform mimics macrophage–lipoprotein interactions, a hallmark of atherosclerotic lesions. MLP-NVs show enhanced accumulation in atherosclerotic plaques, outperforming non-biomimetic nanoparticles. SPMs could also be loaded in this vesicle to promote inflammation resolution in cancer to mimic macrophage–cell debris interaction post chemotherapy.

### 4.3. PLGA (Poly(lactic-co-glycolic acid))

**Figure 2:**
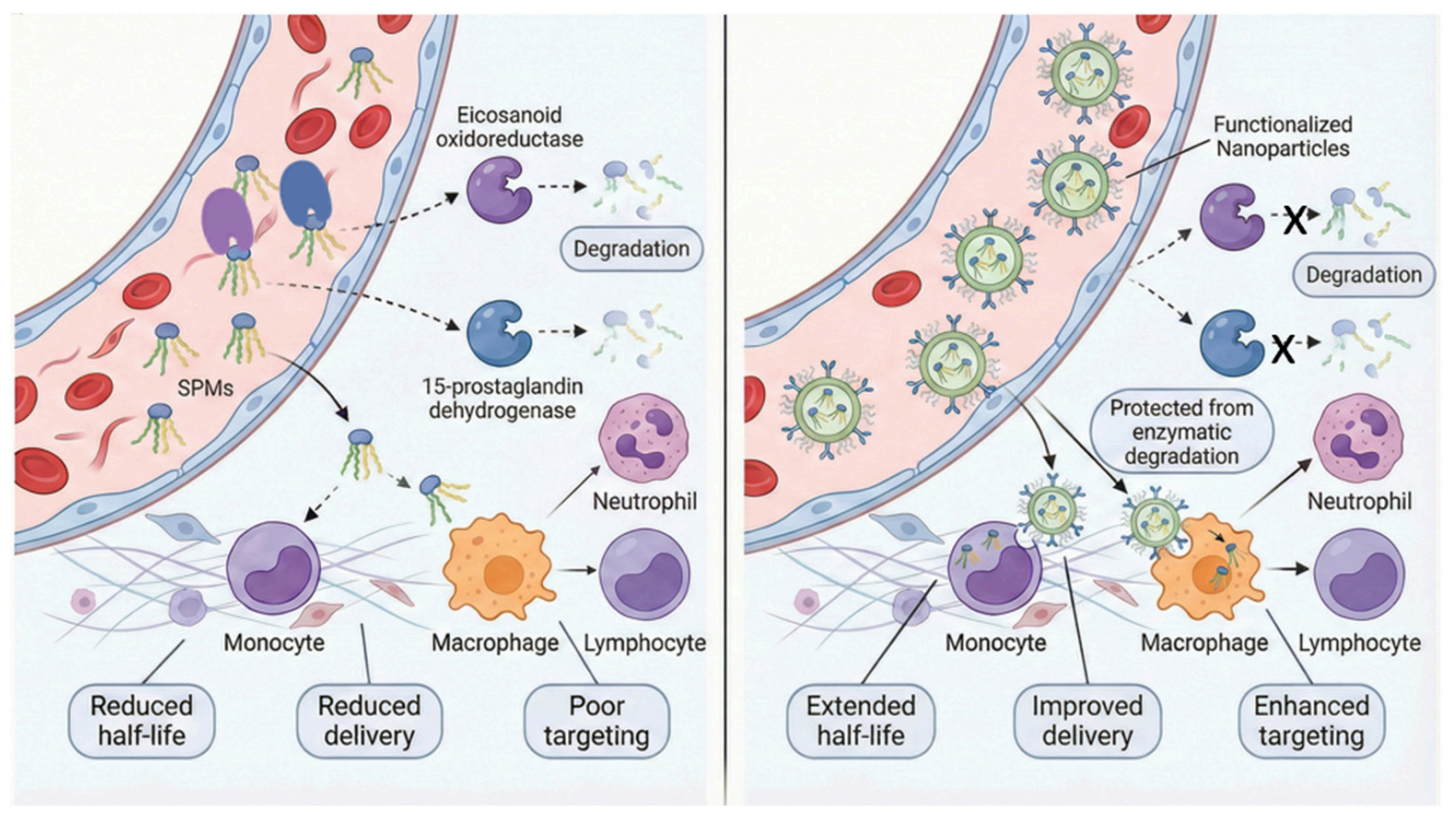
Delivery of SPMs and targeting immune cells with functionalized nanoparticles. The left figure depicts the susceptibility of free SPMs to degradation by *eicosanoid oxidoreductase* and *15*-*prostangladin dehydrogenase* leading to reduced half-life, reduced delivery efficiency and reduced targeting of immune cells within blood and tissue space. The right figure depicts that SPMs delivered by NPs improve their stability, PK and tissue targeting. Figurelabs.ai was used to generate this figure.

Aspirin-triggered RvD1 incorporated into PLGA nanoparticles enhances localized delivery of AT-RvD1 to reduce neutrophil infiltration and promote M2 macrophages and Ly6C^low^ macrophages, which are anti-inflammatory in a model of sterile inflammation [105]. The study showed that the sustained delivery of AT-RvD1 using PLGA film at a cumulative 10 ng dosage over 3 days resolved local inflammation better than the one-time 100 ng dosing of free AT-RvD1. Another study found tissue factor-targeted PLGA nanoparticles can home to injured arteries and deliver synthetic resolvins with high precision [106]. The result showed reduced inflammation and enhanced resolution. Endothelial binding was important for localization, so it would be important to know the major cell types that internalized the particles. While the encapsulation efficiency in the study was 25%, the amount of Resolvins delivered was still sufficient for improved efficacy. Recently, a pH-sensitive smart PLGA-Eudragit 100 nanoparticle was developed to release RvE1 in an alkaline environment caused by biodegradable implants like AZ31 magnesium alloys in rats to promote macrophage mediated inflammation resolution [107]. The common theme in the studies using PLGA to deliver SPMs is local administration of the film or emulsion. Intravenous delivery of such preparation to promote target tissue accumulation would be challenging as the liver would take up most of the particles. The choice of a delivery platform to deliver SPMs depends on the goal of the study. Figure 2 below highlights the benefits of using a delivery platform like nanoparticles to deliver SPMs. Other delivery platforms that have been used to deliver SPMs are hydrogels, emulsion-loaded hydrogels, and black phosphorus nanosheets as listed in the Table 3 [56,108,109]. An emerging strategy for delivery is the chemical conjugation of drug molecules to lipid materials used in formulations as seen in the paclitaxel–sphingomyelin liposomes developed by the Jianqin Lu group [110].

Overall, majority of the delivery platforms for SPMs have enabled active tissue targeting through inherent expression of proteins (Beta-2 integrin as in exosomes) or functionalization of the delivery system with specific peptides/proteins or glycan [75,76,111]. This eventually leads to passive delivery to the cells within the tissue space with inflamed endothelium. Furthermore, the delivery system can be functionalized using glycans such as mannose which targets the CD206 mannose receptor on macrophages [111]. Song et al. showed specific targeting of mouse bone marrow-derived macrophages (BMDMs) using mannose and an N-acetylglucosamine-derived glycopolymer [112]. Uptake of mannosylated liposomes were also shown to improve compared to non-functionalized liposome in rat alveolar macrophages both in vitro and in vivo [111,113]. It was emphasized that the target ligand must be specific for the macrophage of interest, giving the different subtypes and relative populations in different pathologies compared to healthy subjects. This also applies to the other immune cells to reduce the possibility of off-targets effect and side effects.

Table 3 below highlights the receptor-mediated approaches that have been employed to target the different cell types. In the context of addressing the delivery challenges associated with SPMs, functionalizing liposomes or extracellular vesicles with specific proteins or glycan as listed could be a promising strategy.

## 5. Challenges and Perspectives

Specialized pro-resolving mediators (SPMs) represent a mechanistically distinct approach to controlling inflammation because they promote endogenous resolution programs rather than broadly suppressing inflammatory signaling [3,6]. However, despite substantial evidence of biological activity in preclinical models, their therapeutic translation remains at an early stage as they have not been approved clinically. The efficacy observed in experimental models may not be interpreted as evidence that SPMs are established clinical alternatives to corticosteroids or non-steroidal anti-inflammatory drugs. Important uncertainties remain regarding pharmacokinetics, metabolic stability, dose-response relationships, tissue targeting/distribution, receptor pharmacology, and long-term safety in humans. These limitations are related to the properties of SPMs because SPMs are rapidly metabolized and their biological effects are highly dependent on cell types and disease contexts [33,35].

### 5.1. Pharmacokinetics and Pharmacodynamics of SPMs

One of the major barriers to the therapeutic application of SPMs is the discrepancy between their potent biological activity in vitro and unfavorable drug-like properties. As discussed above, SPMs are susceptible to enzymatic inactivation and degradation [35]. Consequently, the concentration of an administered SPM measured in plasma may not adequately represent the amount of biologically active mediator reaching relevant cells within inflamed tissues. This is related to the pharmacokinetics and pharmacodynamics of SPMs. Future studies should incorporate quantitative pharmacokinetic/pharmacodynamic analyses combined with lipidomics to measure administered SPMs and their metabolites in plasma and target tissues.

To increase the pharmacokinetics and pharmacodynamics of SPMs, we need to develop innovative approaches. Antibody–drug conjugates (ADCs) provide a useful conceptual framework for highly tissue-selective delivery because they combine the antigen-recognition properties of monoclonal antibodies with a cleavable therapeutic payload [114]. ADCs can deliver drugs to specific cell types and tissues and increase drug circulation time in vivo. ADCs are well established to treat cancer by delivering potent chemo drugs to tumor cells [115,116,117], but they are less studied in inflammatory diseases. We have shown in Table 4 that immune cells and endothelial cells highly express unique cellular surface molecules, thus we could develop antibody conjugates with SPMs to target immune cells or vasculature related to inflammatory diseases and cancer. For example, endothelial cells highly express ICAM-1 during inflammation responses. We could develop a conjugation strategy to link SPMs to anti-ICAM-1, thus specifically delivering SPMs into inflammatory vasculatures. Using this approach, it is possible to increase pharmacokinetics and pharmacodynamic of SPMs for improved therapeutic effects in treatment of inflammatory diseases and cancer.

### 5.2. Challenges in SPM Delivery Systems

Liposomes, extracellular-vesicles, polymeric nanoparticles, hydrogels, and other biomaterial-based formulations have demonstrated that delivery systems can protect SPMs from degradation, prolong local exposure, and improve therapeutic efficacy relative to free SPM administration in selected preclinical models [56,75,97,98,105,106]. Nevertheless, the current evidence is that predominantly preclinical, and substantial differences in formulation compositions, administration routes, loading efficiency, and release profiles limit direct comparison among platforms. As summarized in Table 3, reported loading efficiencies and release kinetics vary considerably, and the release is frequently characterized in simplified buffers that do not mimic in vivo environments.

Optimized accumulation of a formulation within a targeted organ also does not necessarily establish delivery to the therapeutically relevant cell populations because of complex cell types and the vascular barrier. Another issue is that systemically administered nanoparticles may undergo substantial uptake by the mononuclear phagocyte system, particularly in the liver and spleen. Future studies should therefore distinguish tissue targeting from cell-specific targeting and identify which cell populations internalize the carriers and receive pharmacologically active concentrations of SPMs. Head-to-head studies between free SPMs, non-targeted formulations, and actively targeted formulations will be particularly important for determining whether ligand-mediated targeting provides a meaningful advantage beyond the pharmacokinetic changes produced by encapsulation alone.

This distinction is especially important for the targeting approaches summarized in Table 4. The markers such as ICAM-1, VCAM-1, selectins, beta-2 integrins, and CD206 provide potential routes for targeting inflammatory endothelium or selected immune-cell populations [110,111,112,113,118,119,120,121,122,123,124,125]; however, their expression is not exclusive to diseased tissues and may vary with disease stage and cell phenotypes. Target selection should therefore be informed by quantitative receptor expression, accessibility, and disease-specific cellular distribution rather than simply by the presence of a marker.

The amphiphilic structures of RvD1 and RvD2 create additional formulation challenges because these mediators may partition between aqueous and lipid phases and exhibit limited retention within conventional liposomal formulations [31,96,97,98]. One potential strategy is covalent attachment of an SPM to a lipid component or other carrier-associated molecules to improve formulation retention. For example, conjugation through the carboxyl group of RvD1 to a lipid anchor could theoretically enhance incorporation into lipid bilayers.

Chemical modification of SPMs presents an additional pharmacological challenge. The stereochemistry and positions of functional groups within Resolvins contribute to their receptor interactions and biological activity [96]. Consequently, conjugation chemistry must preserve the pharmacophore or incorporate a linker capable of releasing the native mediator at the target site. Future studies should evaluate not only loading efficiency and release kinetics but also chemical integrity, receptor activation, and pro-resolving activity after release. Development of metabolically stable SPM analogues represents an alternative and complementary strategy that may reduce reliance on complex delivery vehicles [34,128,129].

Recently, Li et al. demonstrated the feasibility of an engineered probiotic (non-pathogenic *E. coli*) expressing *cyclooxygenase (COX-2)* and *lipoxygenase (5-LOX)* enzymes to sustainably produce RvE1 in situ to treat colitis and acute inflammation [130]. Although the approach did not address the rapid enzymatic degradation and short half-life of RvE1, the long-term persistence of the bacteria following oral administration facilitated sustained biosynthesis. While the development is still in its early stage, the vast opportunities for SPM therapeutics are becoming increasingly explored.

### 5.3. Biological Complexity and Disease-Specific Responses

Another challenge is that resolution is not mediated by a single cell population or signaling pathway. SPMs act within networks involving neutrophils, monocytes, macrophages, lymphocytes, dendritic cells, and endothelial cells, and the relative contribution of these populations varies according to diseases and stages. Furthermore, receptors such as ALX/FPR2 can interact with multiple ligands and participate in different inflammatory contexts [44,45]. Receptor expression alone may therefore be insufficient to predict therapeutic responsiveness, emphasizing the need to define the relevant target cells, receptor states, and inflammatory microenvironment for each indication.

This complexity is particularly relevant in cancer. Although SPMs have suppressed tumor growth or therapy-associated tumor regrowth in several experimental systems [4,54,58,80,90], the evidence remains heterogeneous and is insufficient to establish SPMs as standalone anticancer therapeutics. As discussed in Section 3, differences between tumor models, treatment combinations, and the presence of therapy-induced apoptotic or necrotic materials can substantially alter the observed response. SPMs may ultimately be more useful in defined therapeutic contexts, for example as adjuncts following chemotherapy, radiotherapy, or surgery, than as direct tumoricidal agents. This possibility requires systematic testing across immunocompetent tumor models and, subsequently, human studies.

## 6. Conclusions

SPMs have compelling preclinical pro-resolving activity across inflammatory disease and cancer models, while existing delivery platforms demonstrate that formulations can improve stability, localization, and therapeutic performance in selected settings. However, clinical translation remains limited by incomplete pharmacokinetic and pharmacodynamic characterization, chemical and metabolic instability, uncertain target-cell(s) exposure, disease-dependent biology, and the lack of standardized comparisons among delivery strategies. Future development should therefore prioritize mechanistically informed targeting, quantitative biodistribution and target-engagement studies, rigorous comparison with free SPMs, and scalable formulations. Emerging concepts such as lipid conjugation and antibody-directed delivery are promising approaches, but they should be experimentally validated.

Ultimately, the key question is not simply whether a delivery platform can increase SPM circulation time or tissue accumulation, but whether it can deliver an appropriate concentration of biologically active mediator to the relevant cell population at the appropriate stage of disease. Resolving this relationship between formulations, pharmacokinetics, cellular targeting, and pharmacodynamic response will be essential for determining whether the substantial biological promise of SPMs can be translated into clinically useful therapies.

## Figures and Tables

**Table 1:** Effects of SPMs in inflammatory diseases.

Diseases	SPMs	Cell Types	Receptors	Outcomes
Murine atherosclerosis	RvD1-100 ng/mouse twice per week via osmotic pump	Macrophages	ALX/FPR2	Reduced plaque formation and stability [56]
Brain ischemia reperfusion injury	RvD2	Neutrophils	GPR18	Smaller infarct volumes, improved neurological scores [75]
*P. aeruginosa*-induced lung inflammation	RvD1-67 ng/mouse	Neutrophils	ALX/FPR2	Enhanced inflammation resolution and bacteria killing [77]
Cigarette smoke impacting COPD prognosis	RvD1-1-100 nM (in vitro)	Macrophage	GPR32	Reversal of macrophage dysfunction [55]
	RvD2-1-100 nM (in vitro)		GPR18	
Amyotrophic lateral sclerosis (ALS)	RvD1–2.6 nM-26.6 nM (in vitro)	Macrophages	GPR32	Inhibition of inflammatory macrophage-mediated neuronal phagocytosis [57]
LPS-induced cardiac injury	RvD1–5 ug/kg intraperitoneally	Macrophages	ALX/FPR2	Reduction in myocardial inflammation [95]

**Table 2:** Effects of SPMs in cancer.

Pathology	Used Drugs	SPMs	Cell Type	Target Receptor	Outcome
Breast Prostate Cancer	–	DHA—5 g/day orally EPA—1.8 g per day orally	Tumor cells	–	Reduction in Ki67 and reduction in tumor growth [88,89]
Chemotherapy-induced cancer regrowth	–	RvD1, RvD2—15 ng/day via osmotic pump	Macrophages	ALX/FPR2, GPR18	Inhibition of tumor growth [4]
Melanoma	Doxorubicin	RvD2—100 ng/mouse intratumorally 3 times a week	Macrophages neutrophils	GPR18	Reduced tumor growth, improved survival (not yet published)
Lung adenocarcinoma	–	RvD1—100 nM (in vitro)	Cancer cells (A549)	GPR32	Reduced epithelial–mesenchymal transition [90]
Human papilloma virus-positive cancer (Hela)	–	RvD1—100 ng intraperitoneally 3 times per week	Neutrophils Monocytes	GPR32	Increased recruitment of antitumor monocyte [80]

**Table 3:** Existing delivery system for SPMs, loading efficiency, and drug release time.

Delivery System	SPM	Encapsulation efficiency ^a^, Loading Efficiency ^b^ Loading Capacity ^c^ (%)	Drug Release Time (50–90%)	Observation
Liposomes	RvD1	71 ^a^, 55 ^a^	11 days	Drug release time investigated in phosphate-buffered saline (PBS) in vitro [97,98].
Extracellular vesicles (EVs)	RvD2	10 ^b^	24 h	Drug release investigated in HBSS and 20% serum [75].
Nanosheets	RvD1	0.02 ^c^	3.5 h	Drug release carried out in a solution with pH 6.3 + 1.0 mM H_2_O_2_ [56].
PLGA	RvD1	25 ^a^	4 days	Drug release carried out in phosphate-buffered saline (PBS) [105].
Hydrogel	RvD1	–	18 days	Drug release study in PBS [56].
Emulsion-loaded hydrogel	LXA4	26 ^a^	3 days	Drug release study in hyaluronidase solution 50 U/ml [109].
Gel Methacrylate	RvD1	–	15 days	Drug release study in PBS [108].

**Table 4:** Targeting strategies for specific cell types, ligands, and the delivery systems used in different pathologies.

Cell Type	Target Receptor/Marker	Targeting Moiety/Strategy	Example of Delivery Systems	Outcomes
Macrophages	CD206 (mannose receptor)	Mannose/mannosylated ligands	Mannosylated liposomes/polymeric NPs for CD206^+^ macrophages	Improved delivery to tissue associated macrophages to improve therapeutic action in glioblastoma [118].
	CD206 (TAMs)	CD206-binding peptide (e.g., CSPGAKVRC)	Peptide-decorated NPs targeting tumor-associated macrophages	Precision targeting of TAMs within tumor microenvironment [119].
	Scavenger receptors (SR-A, SR-B1)	23-mer poly-Guanine (polyG), microRNA-146a oligonucleotides	SR-targeted NPs for plaque macrophages	Reduced atherosclerotic plaque with limited toxicity and foam cells [120,121].
Neutrophils	β2 integrins (CD11b/CD18)	ICAM-1-derived peptides/antibodies	ICAM-binding nanoparticles, e.g., exosomes adhering to activated neutrophils and endothelium	Liposomes trafficked pulmonary-induced leukocytes like neutrophils to cross blood–brain barrier for drug delivery [122]. Reduced brain ischemia score [75].
Endothelial cells	VCAM-1	VHPKQHR peptide	VCAM-1-targeted NPs for atherosclerotic endothelium	In vivo studies confirmed that CAM-targeted nanoparticles accumulate at sites of atherosclerotic lesions and vascular inflammation [123].
	ICAM-1	Anti-ICAM-1 antibodies/peptides	ICAM-1-targeted liposomes/Nanostructured Lipid carriers NLCs for inflamed endothelium	NLC-targeted system improves therapeutic efficacy in acute lung injury models with inflamed endothelia compared to non-targeted formulations [124].
	E-/P-selectin	Selectin-binding peptide, e.g., CGGSSLVSVLDLEPLDAAWL	Selectin-targeted NPs for inflamed vasculature	Targeted nanoemulsions of dexamethasone successfully bind to P-selectin-expressing endothelial cells to reduce inflammation [125].
	Damaged endothelium/thrombus	Platelet membrane coating	Platelet-mimetic NPs homing to injured vessels and plaques	Platelet-like features of the nanoparticles significantly improve targeting efficiency to sites of vascular damage [126].
Dendritic cells	C-type lectins/TLRs	Glycans + CpG, poly(I:C)	DC-targeted immunostimulatory NPs (antigen + adjuvant co-delivery)	Improved lymphatic delivery of nucleic acid polyinosinic:polycytidylic acid (PIC) [127].

## Data Availability

No new data were created or analyzed in this study. Data sharing is not applicable to this article.

## Abbreviations

The following abbreviations are used in this manuscript:

AA	Arachidonic acid
ALX/FPR2	Lipoxin A4 receptor/Formyl peptide receptor 2
ALS	Amyotrophic lateral sclerosis
AMPK	AMP-activated protein kinase
ARDS	Acute respiratory distress syndrome
AT-RvD1	Aspirin-triggered resolvin D1
BLT1	Leukotriene B4 receptor 1
CD14	Cluster of differentiation 14
CD69	Cluster of differentiation 69
CD206	Cluster of differentiation 206
C5aR1	Complement component 5a receptor 1
ChemR23/ERV1	Chemerin receptor 23
CNS	Central nervous system
COPD	Chronic obstructive pulmonary disease
CXCR1	C-X-C motif chemokine receptor 1
CXCR2	C-X-C motif chemokine receptor 2
DDAB	Dimethyldioctadecylammonium bromide
DHA	Docosahexaenoic acid
DOPG	1,2-Dioleoyl-sn-glycero-3-phosphoglycerol
DRV1/GPR32	G-protein receptor 32
DRV2/GPR18	G-protein receptor 18
ELISA	Enzyme-linked immunosorbent assay
EMT	Epithelial-mesenchymal transition
EPA	Eicosapentaenoic acid
EVs	Extracellular vesicles
FFAR4/GPR120	Free fatty acid receptor 4
GPR18	G-protein receptor 18
GPR32	G-protein receptor 32
GM-CSF	Granulocyte-macrophage colony-stimulating factor
GPCR	G-protein coupled receptor
HBSS	Hank’s balanced salt solution
H_2_O_2_	Hydrogen peroxide
ICAM-1	Intercellular adhesion molecule 1
IL-1β	Interleukin-1 beta
IL-6	Interleukin-6
IL-8	Interleukin-8
IL-10	Interleukin-10
IL-12	Interleukin-12
IL-17	Interleukin-17
IL-21	Interleukin-21
IL-22	Interleukin-22
IL-23	Interleukin-23
LBP	Lipopolysaccharide-binding protein
MaRs	Maresins
MLPNVs	Macrophage-lipidated peptide nanovesicles
MMAE	Monomethyl Auristatin E
MPO	Myeloperoxidase
NETs	Neutrophil extracellular traps
NF-κB	Nuclear factor kappa-B
NLCs	Nanostructured lipid carriers
NPs	Nanoparticles
PAFR	Platelet-activating factor receptor
PBS	Phosphate-buffered saline
PDs	Protectins
PIC	Polyinosinic:polycytidylic acid
PLGA	Poly(lactic-co-glycolic acid)
PM A	Phorbol 12-myristate 13-acetate
PS	Phosphatidylserine
PUFAs	Polyunsaturated fatty acids
RvD1	Resolvin D1
RvD2	Resolvin D2
RvEs	Resolvin E-series resolvins
RvTs	Resolvin T-series
ROS	Reactive oxygen species
RORγ	Retinoic acid-related orphan receptor gamma
SPMs	Specialized pro-resolving mediators
TAMs	Tumor-associated macrophages
TANs	Tumor-associated neutrophils
Th1	T helper 1 cell
Th2	T helper 2 cell
Th17	T helper 17 cell
TLR4	Toll-like receptor 4
TNF-α	Tumor necrosis factor alpha
Treg	Regulatory T cell
VCAM-1	Vascular cell adhesion molecule 1
